# Persistent spin texture enforced by symmetry

**DOI:** 10.1038/s41467-018-05137-0

**Published:** 2018-07-17

**Authors:** L. L. Tao, Evgeny Y. Tsymbal

**Affiliations:** Department of Physics and Astronomy, Nebraska Center for Materials and Nanoscience, University of Nebraska, Lincoln, NE 68588 USA

## Abstract

Persistent spin texture (PST) is the property of some materials to maintain a uniform spin configuration in the momentum space. This property has been predicted to support an extraordinarily long spin lifetime of carriers promising for spintronics applications. Here, we predict that there exists a class of noncentrosymmetric bulk materials, where the PST is enforced by the nonsymmorphic space group symmetry of the crystal. Around certain high symmetry points in the Brillouin zone, the sublattice degrees of freedom impose a constraint on the effective spin–orbit field, which orientation remains independent of the momentum and thus maintains the PST. We illustrate this behavior using density-functional theory calculations for a handful of promising candidates accessible experimentally. Among them is the ferroelectric oxide BiInO_3_—a wide band gap semiconductor which sustains a PST around the conduction band minimum. Our results broaden the range of materials that can be employed in spintronics.

## Abstract (web summary)

Persistent spin texture (PST) can generate fascinating physics that is promising for spintronics applications but requires non-trivial sample design. Here the authors alternatively propose that a class of materials has intrinsic PST enforced by the nonsymmorphic space group symmetry of the crystal.

## Introduction

In recent years, there has been increasing interest in materials and structures, where quantum effects are responsible for novel physical properties, revealing the important roles of symmetry, topology, and dimensionality^1^. Among such quantum materials are graphene, topological insulators, Weyl semimetals, and superconductors. In many cases, the quantum materials derive their properties from the interplay between the electron, spin, lattice, and orbital degrees of freedom, resulting in complex physical phenomena and emergent functionalities^2^. These new functionalities are interesting due to their potential for a continuously evolving field of spintronics^3^.

**Fig. 1:**
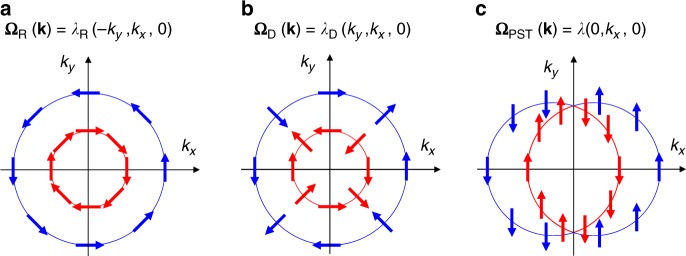
Spin texture. **a**–**c** Spin structure resulting from spin-orbit coupling in a system lacking an inversion center: Rashba (**a**), Dresselhaus (**b**), and persistent spin texture (**c**) configurations. Blue and red arrows indicate spin orientation for the two electronic subbands resulting from SOC. Expressions for the respective SOC fields ${\mathbf{\Omega }}({\mathbf{k}})$ are shown. Note that ${\mathbf{\Omega }}_{\mathrm{D}}$ is represented in the coordinate system with the *x*- and *y*-axes being perpendicular to the mirror planes of an orthorhombic system (*M*_*x*_ and *M*_*y*_ in Fig. 2a)

Regarding the new phenomena, often a special role is played by the spin–orbit coupling (SOC), which on its own has inspired a vast number of predictions, discoveries, and novel concepts^4^. In a system, lacking an inversion center, the SOC results in an effective momentum-dependent magnetic field acting on spin **σ**. This field **Ω**(**k**) is odd in the electron’s wave vector (**k**), as was first demonstrated by Dresselhaus^5^ and Rashba^6^, so that the effective SOC Hamiltonian can be written as

1 $$H_{{\mathrm{SO}}} = {\mathbf{\Omega }}({\mathbf{k}}) \cdot {\mathbf{\sigma }},$$

preserving the time-reversal symmetry. The specific form of **Ω**(**k**) depends on the space symmetry of the system. For example, in case of the *C*_2v_ point group, the Dresselhaus and Rashba SOC fields can be written as ${\mathbf{\Omega }}_{\mathrm{D}}({\mathbf{k}}) = \lambda _{\mathrm{D}}(k_y,k_x,0)$ and ${\mathbf{\Omega }}_{\mathrm{R}}({\mathbf{k}}) = \lambda _{\mathrm{R}}( - k_y,k_x,0)$, respectively. Such SOC leads to a chiral spin texture of the electronic bands in the momentum space, as shown in Fig. 1a, b. The chiral spin textures driven by the SOC can be exploited to create nonequilibrium spin polarization^7^, produce the spin Hall effect^8^, and design a spin field-effect transistor (FET)^9^. Recently, these and other related phenomena have received significant attention and led to the emergence of a new field of research—spin-orbitronics^4^.

Although large SOC is beneficial for realizing these phenomena, it plays a detrimental role for the spin life time. In a diffusive transport regime, impurities and defects scatter electrons, changing their momentum and randomizing the spin, due to the momentum-dependent spin–orbit field ${\mathbf{\Omega }}({\mathbf{k}})$. This process is known as the Dyakonov-Perel spin relaxation^10^, reduces the spin life time and, thus limits the performance of potential spintronic devices, e.g., the spin FET. A possible way to circumvent this effect is to engineer a structure, where the spin–orbit field orientation is momentum-independent^11^. This can be achieved, in particular, if the magnitudes of $\lambda _{\mathrm{R}}$ and $\lambda _{\mathrm{D}}$ are equal, i.e., $\lambda /2 = \lambda _{\mathrm{D}} = \pm\lambda _{\mathrm{R}}$, resulting in a unidirectional spin–orbit field, ${\mathbf{\Omega }}_{{\mathrm{PST}}} = \lambda (k_y,0,0)$ or ${\mathbf{\Omega }}_{{\mathrm{PST}}} = \lambda (0,k_x,0)$, and thus a momentum-independent spin configuration, known as the persistent spin texture (PST) (Fig. 1c)^12^.

Under these conditions, electron motion is accompanied by spin precession around the unidirectional spin–orbit field, resulting in a spatially periodic mode known as a persistent spin helix (PSH)^13^. The PSH state arises due to the SU(2) spin rotation symmetry, which is robust against spin-independent disorder and renders an ultimately infinite spin lifetime^14^. The PSH has been experimentally demonstrated in the two-dimensional electron gas semiconductor quantum-well structures, such as GaAs/AlGaAs^15,16^ and InGaAs/InAlAs^17,18^, where the required condition of equal Rashba ($\lambda _{\mathrm{R}}$) and Dresselhaus ($\lambda _{\mathrm{D}}$) parameters was realized through tuning the quantum-well width, doping level, and applied external electric field.

Despite these advances, a number of difficulties impede the practical application and further experimental studies of these semiconductor heterostructures. Satisfying the stringent condition of equal $\lambda _{\mathrm{R}}$ and $\lambda _{\mathrm{D}}$ parameters is technically nontrivial because it requires a precise control of the quantum-well width and the doping level. Furthermore, due to the small values of these parameters (a few meV Å), efficient spin manipulation by an applied electric field is questionable. Recently, based on first-principles calculations a PST was predicted for a wurtzite ZnO $(10\bar 10)$ surface^19^ and a tensile-strained LaAlO_3_/SrTiO_3_ (001) interface^20^. However, for the latter, too large tensile strain (>5%) is required to achieve the desired property, whereas for the former, the SOC energy splitting is too small (~1 meV). It would be desirable to find bulk materials where the PST is a robust intrinsic bulk property. Recently, SnTe (001) thin films have been proposed to realize a PSH^21^.

Here, we propose a conceptually different approach to achieve the PST. We demonstrate that there exist a class of noncentrosymmetric bulk materials where the PST is enforced by nonsymmorphic space group symmetry of the crystal, i.e., the space group combining point-group symmetry operations with nonprimitive translations^22^. Around certain high symmetry points in the Brillouin zone, the sublattice degrees of freedom impose a constraint on the effective spin–orbit field, which orientation remains independent of the momentum and thus maintains PST. The symmetry-enforced PST survives over the large part of the Brillouin zone including band edges, as we demonstrate using density-functional theory (DFT) calculations for BiInO_3_ and other materials with appropriate crystal group symmetry.

## Results

### Symmetry analysis

**Fig. 2:**
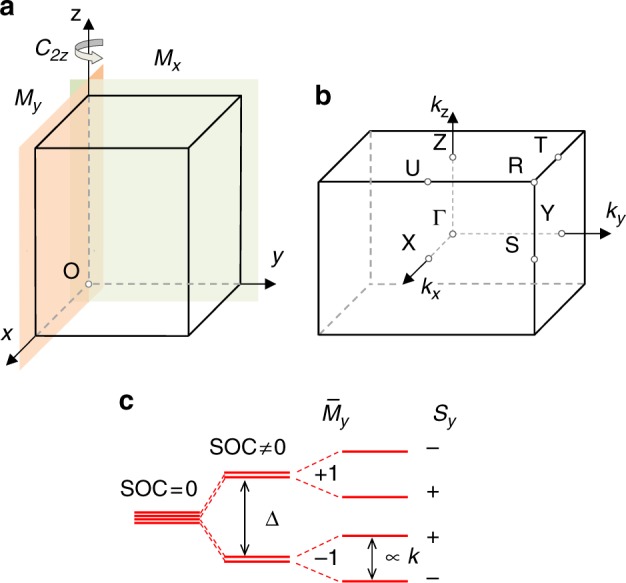
Crystal lattice and energy band splitting. **a** Orthorhombic crystal lattice with symmetry operations indicated. $C_{2z}$ denotes a twofold rotation operator, and $M_x$ and $M_y$ represent two mirror reflection operators. **b** The first Brillouin zone with the high symmetry k points indicated: Γ (0, 0, 0), X (π, 0, 0), S (π, π, 0), Y (0, π, 0), Z (0, 0, π), U (π, 0, π), R (π, π, π), and T (0, π, π), the k point coordinates are given in units of the reciprocal lattice constants. **c** Schematic splitting of the energy levels around the X point. SOC splits the state into two doublets with eigenvalues of $\bar M_y = \pm1$, which are further split into singlets with sign-reversed expectation values of $s_y$. The energy level order labeled by $\bar M_y$ and $s_y$ is material dependent

We consider orthorhombic nonsymmorphic crystals with broken space inversion symmetry (space groups listed in Table 1)^22^. Figure 2a shows an orthorhombic crystal lattice, which contains the following symmetry operations: (1) the identity operation *E*; (2) glide reflection $\bar M_x$, which consists of mirror reflection about the *x* = 0 plane $M_x$ followed by the $(\mu _1\,\nu _1\,\eta _1)$ translation:

2 $$\bar M_x:(x,y,z) \to ( - x + \mu _1,y + \nu _1,z + \eta _1),$$

**Table 1:** Classification of orthorhombic space groups with no inversion symmetry according to translation vectors characterized by indices (*μ*_2_*v*_1_). Nonzero spin components in high symmetry points and band degeneracy along high symmetry lines are shown

(*μ*_2_ ν_1_)	X	Y	Band degeneracy	Space group no.
$({\textstyle{1 \over 2}}0)$	$s_y$	–	X–S	28, 29, 31, 40, 46
$(0{\textstyle{1 \over 2}})$	–	$s_x$	Y–S	30, 39
$({\textstyle{1 \over 2}}{\textstyle{1 \over 2}})$	$s_y$	$s_x$	X–S and Y–S	32, 33, 34, 41, 45

(3) glide reflection $\bar M_y$, which consists of mirror reflection about the *y* *=* 0 plane $M_y$ followed by the $(\mu _2\,\nu _2\,\eta _2)$ translation:

3 $$\bar M_y:(x,y,z) \to (x + \mu _2, - y + \nu _2,z + \eta _2),$$

(4) two fold screw rotation $\bar C_{2z}$, which consists of twofold rotation around the *z*-axis $C_{2z}$ followed by the $(\mu _3\,\nu _3\,\eta _3)$ translation:

4 $$\bar C_{2z}:(x,y,z) \to ( - x + \mu _3, - y + \nu _3,z + \eta _3).$$

Here and below, the translation and reciprocal vectors are given in units of lattice constants and $\mu _i,\nu _i,\eta _i = 0,\,\,{\textstyle{1 \over 2}}$ (*i* *=* 1, 2, 3). The glide (screw) symmetry is reduced to mirror (rotation) symmetry if $\mu _i = \nu _i = \lambda _i = 0$. In addition, we assume that the system exhibits time-reversal symmetry *T*.

Now, we demonstrate the formation of the PST around the X point ${\mathbf{k}} = ({\mathrm{\pi }},0,0)$ in the Brillouin zone of the crystal (Fig. 2b). First, we consider the X–S high symmetry line ${\mathbf{k}} = ({\mathrm{\pi }},k_y,0)$. Along this line, the little group of wave vector **k** includes the symmetry operators $\bar M_x$ and $\Theta \equiv T\bar M_y$, as follows from $k_y$ being invariant under the transformations determined by these symmetry operators. Since $T^2 = - 1$ for a spin-half system, we find $\Theta ^2 = T^2\bar M_y^2 = {\mathrm{e}}^{ - 2{\mathrm{i}}\mu _2\pi }$. Therefore, for the space groups with $\mu _2 = {\textstyle{1 \over 2}}$, along the X–S line, $\Theta ^2 = - 1$ so that all bands are double degenerate. The doublet states $(\psi _{\mathbf{k}},\,\Theta \psi _{\mathbf{k}})$ form a Kramers pair.

At the X point, $\bar M_y$ commutes with the Hamiltonian of the crystal, i.e., $[ {\bar M_y,H}] = 0$, and the doublet $(\psi _{\mathrm{X}},\,\Theta \psi _{\mathrm{X}})$ can be labeled using the eigenvalues of $\bar M_y$. Since $\bar M_y^2 = 1$ at this point, we have $\bar M_y\psi _{\mathrm{X}}^ \pm = \pm \psi _{\mathrm{X}}^ \pm$ and $\bar M_y\Theta \psi _{\mathrm{X}}^ \pm = \pm \Theta \psi _{\mathrm{X}}^ \pm$. Thus, by symmetry, there are two conjugated doublets at the X point, $(\psi _{\mathrm{X}}^ + ,\,\Theta \psi _{\mathrm{X}}^ + )$ or $(\psi _{\mathrm{X}}^ - ,\,\Theta \psi _{\mathrm{X}}^ - )$, which are distinguished by the $\bar M_y$ eigenvalues. Within each of the two doublets, matrix elements of the spin operators $\sigma _x$ and $\sigma _z$ are equal to zero. This is due to the fact that in the spin space $\bar M_y$ anticommutes with $\sigma _x$ and $\sigma _z$, i.e., $\left\{ {\bar M_y,\sigma _{x,z}} \right\} = 0$, which results in $\left\langle {\psi _{\mathrm{X}}^ + } \right|\sigma _{x,z}\left| {\psi _{\mathrm{X}}^ + } \right\rangle = \left\langle {\psi _{\mathrm{X}}^ + } \right|\bar M_y^{ - 1}\sigma _{x,z}\bar M_y\left| {\psi _{\mathrm{X}}^ + } \right\rangle = - \left\langle {\psi _{\mathrm{X}}^ + } \right|\sigma _{x,z}\left| {\psi _{\mathrm{X}}^ + } \right\rangle$, and hence $\left\langle {\psi _{\mathrm{X}}^ + } \right|\sigma _{x,z}\left| {\psi _{\mathrm{X}}^ + } \right\rangle = 0$. The similar analysis leads to $\left\langle {\Theta \psi _{\mathrm{X}}^ + } \right|\sigma _{x,z}\left| {\Theta \psi _{\mathrm{X}}^ + } \right\rangle = 0$ and $\left\langle {\psi _{\mathrm{X}}^ + } \right|\sigma _{x,z}\left| {\Theta \psi _{\mathrm{X}}^ + } \right\rangle = 0$. The same conclusion holds for the other doublet $(\psi _{\mathrm{X}}^ - ,\,\Theta \psi _{\mathrm{X}}^ - )$.

We see, therefore, that any state, which represents a linear combination of the states comprising either doublet, i.e., $\psi _{\mathbf{k}}^ \pm = a_{\mathbf{k}}\psi _{\mathrm{X}}^ \pm + b_{\mathbf{k}}\Theta \psi _{\mathrm{X}}^ \pm$ (where $a_{\mathbf{k}}$ and $b_{\mathbf{k}}$ are some coefficients), has zero expectation values of $\sigma _{x,z}$ and zero spin components $s_{x,z} = {\textstyle{1 \over 2}}\left\langle {\psi _{\mathbf{k}}^ \pm } \right|\sigma _{x,z}\left| {\psi _{\mathbf{k}}^ \pm } \right\rangle = 0$. The only nonzero component of the spin is, therefore, $s_y$. Thus, as long as the two doublets are not mixed, the spin orientation is forced to be along the *y*-direction.

This explains the PST around the X point. At the X point the SOC splits the fourfold degenerate state into two doublets with splitting *Δ* and eigenvalues of $\bar M_y = \pm 1$, as shown in Fig. 2c. When moving away from this point the perturbation breaks the X point symmetry and further splits the doublets, each into two singlets (unless going along the X–S symmetry line). These states preserve the unidirectional spin texture along the *y*-direction unless the perturbation is so strong that it mixes the doublets. However, due to the perturbation being linear with respect to **k** (measured from the X point), there is always a range of **k** vectors, where it is small compared to the splitting between the doublets. In practice, this range of **k** values may be substantial and can span a large portion of the Brillouin zone including the band edges responsible to transport and optical properties in semiconductor materials.

A similar analysis applies to the Y point, where ${\mathbf{k}} = (0,{\mathrm{\pi }},0)$ (Fig. 2b). The bands are double degenerate along the high symmetry Y–S line, where ${\mathbf{k}} = (k_x,{\mathrm{\pi }},0)$, provided that $\nu _1 = {\textstyle{1 \over 2}}$. The wave functions at the Y point are the eigenstates of the spin component $\sigma _x$. A portion of the Brillouin zone around the Y point maintains the PST with the spin pointing along the *x*-direction. In Table 1 we classify space groups of the orthorhombic crystal system according to the $(\mu _2\,\nu _1)$ value and show those spin components ${\mathbf{s}} = (s_x,s_y,s_z)$ which remain nonzero around the respective high-symmetry points.

### DFT analysis of bulk BiInO_3_

**Fig. 3:**
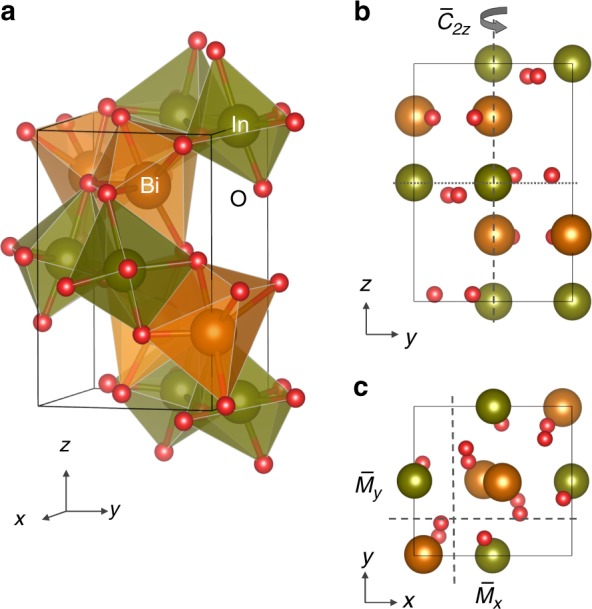
Crystal structure of bulk BiInO_3_. **a** 3D view of the unit cell structure. **b**, **c** View of the crystal structure in the (100) plane (**b**) and the (001) plane (**c**). The twofold screw rotation axis ($\bar C_{2z}$) and the glide reflection planes ($\bar M_x$ and $\bar M_y$) are indicated by the dashed lines. The dotted line indicates a *Pnma* symmetry mirror plane

In the following, we reinforce our symmetry-based conclusions by performing DFT calculations for a number of bulk compounds, which belong to selected space groups listed in Table 1. Details of the DFT calculations are described in Section Methods. First, we focus on perovskite BiInO_3_ (space group No. 33), which has been synthesized experimentally and is stable at ambient conditions^23^. The BiInO_3_ crystal structure (Fig. 3) belongs to the *Pna*2_1_ orthorhombic phase (space group No. 33). The symmetry operations of this group involve the glide reflection $\bar M_x$ (2) with $(\mu _1 = {\textstyle{1 \over 2}},\,\,\nu _1 = {\textstyle{1 \over 2}}\,,\,\,\eta _1 = {\textstyle{1 \over 2}})$, the glide reflection $\bar M_y$ (3) with $(\mu _2 = {\textstyle{1 \over 2}},\,\,\nu _2 = {\textstyle{1 \over 2}}\,,\,\,\eta _2 = 0)$ and the twofold screw rotation $\bar C_{2z}$ (4) with $(\mu _3 = 0,\,\,\nu _3 = 0\,,\,\,\eta _3 = {\textstyle{1 \over 2}})$. The BiInO_3_ crystal structure is derived from the centrosymmetric GdFeO_3_-type perovskite structure (*Pnma* group) through polar displacements, which break space inversion symmetry. As seen from Fig. 3a, each bismuth or indium atom in the BiInO_3_ structure is surrounded by a distorted oxygen octahedron typical for the GdFeO_3_-type perovskite structure. In addition, there are polar displacements seen, e.g., in Fig. 3b from displacement of Bi^3+^ ions (~0.25 Å) from their symmetric positions with respect to the mirror *Pnma* plane (dotted line in Fig. 3b). The polar displacements yield a finite polarization pointing in the [001] direction. There are two topologically equivalent variants of the space group *Pna*2_1_ with opposite polarization (pointing in the $[001]$ or $[00\bar 1]$ directions) indicative to the ferroelectric nature of BiInO_3_. The calculated polarization is about 33.6 μC/cm^2^.

**Fig. 4:**
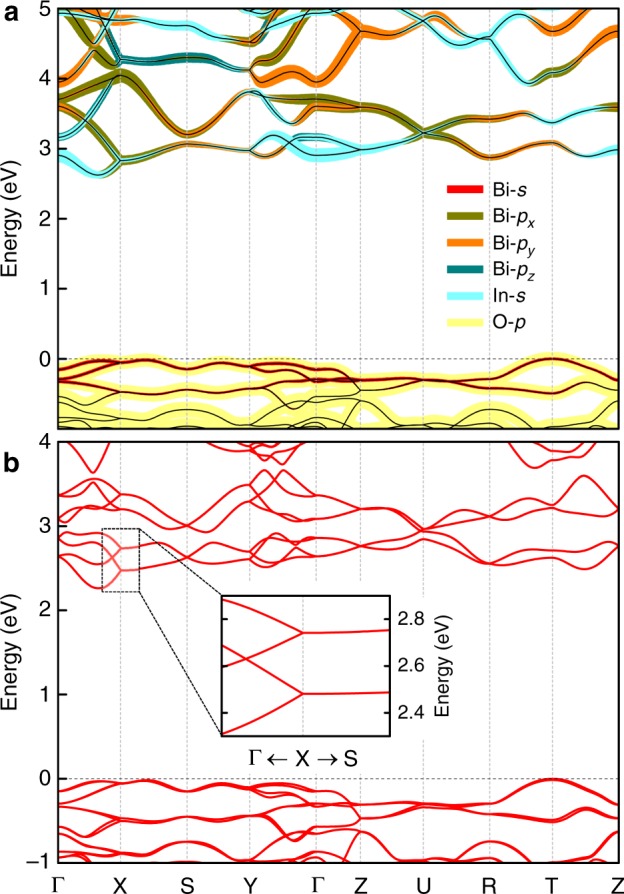
Band structure of bulk BiInO_3_. **a, b** Band structure along the high symmetry lines in the Brillouin zone without SOC (**a**) and with SOC (**b**). Orbital-contributions in panel **a** are shown by color lines with thickness proportional to the orbital weight. Inset in panel **b** shows the band structure zoomed in around the X point

Figure 4a shows the calculated band structure of BiInO_3_ without SOC along high-symmetry lines in the Brillouin zone (shown in Fig. 2b). We find that the conduction bands are mostly composed of the hybridized Bi-6*p* and In-5*s* orbitals, whereas the valence bands are dominated by the O-2*p* orbitals with a small admixture of the Bi-6s states. It is seen from Fig. 4a that BiInO_3_ is an indirect band-gap semiconductor with the valence band maximum located at the T point and the conduction band minimum (CBM) located along the Γ–X symmetry line. The calculated band gap is about 2.6 eV.

Including SOC (Fig. 4b) reduces the band gap to about 2.3 eV and strongly affects the electronic structure of conduction bands of BiInO_3_. Comparing the band structures calculated with SOC (Fig. 4b) and without SOC (Fig. 4a), a sizable band spin splitting produced by the SOC is seen at some high symmetry **k** points and along certain **k** paths. At the X point, which is located in the proximity of the CBM, the two lower energy states are doublets resulting from the SOC splitting. The splitting is large, i.e., *Δ* ≈ 0.26 eV. As expected, the bands along the X–S line are double degenerate protected by the $\Theta$ symmetry. When moving from the X to Γ point the doublets are split into singlets with a nearly linear dispersion (see inset of Fig. 4b).

Lifting the degeneracy along the Γ–X line, where ${\mathbf{k}} = (k_x,0,0)$, can be understood from the little group of wave vector **k**, which has symmetry generators $\bar M_y$ and $\tilde \Theta \equiv T\bar M_x$. Along this line $\tilde \Theta ^2 = T^2\bar M_x^2 = {\mathrm{e}}^{ - {\mathrm{i}}k_y - {\mathrm{i}}k_z} = 1$ and thus the Bloch states $\psi _{\mathbf{k}}$ and $\tilde \Theta \psi _{\mathbf{k}}$ are not degenerate. In addition, each state $\psi _{\mathbf{k}}$ can be labeled using the eigenvalues of $\bar M_y$. Since $\bar M_y^2 = - {\mathrm{e}}^{ - {\mathrm{i}}k_x}$, we obtain $\bar M_y\left| {\psi _{\mathbf{k}}^ \pm } \right\rangle = \pm {\mathrm{ie}}^{ - {\mathrm{i}}{\textstyle{{k_x} \over 2}}}\left| {\psi _{\mathbf{k}}^ \pm } \right\rangle$. Therefore, there are four nondegenerate Bloch states, $\psi _{\mathbf{k}}^ \pm$ and $\tilde \Theta \psi _{\mathbf{k}}^ \pm$, evolving from the X point when moving along the X–Γ line (inset of Fig. 4b). Interestingly, crossing the $\psi _{\mathbf{k}}^ +$ and $\psi _{\mathbf{k}}^ -$ bands is enforced and protected by symmetry, resulting in a hourglass-shaped band dispersion^24,25^ (see Supplementary Note 1).

The SOC splitting at the Y point is smaller *Δ* ≈ 0.09 eV. The bands along the Y–S line are double degenerate protected by the $\tilde \Theta$ symmetry, but split when moving from the Y to Γ point. This behavior can be understood using the considerations similar to those we used to explain band degeneracies and splittings around the X point.

**Fig. 5:**
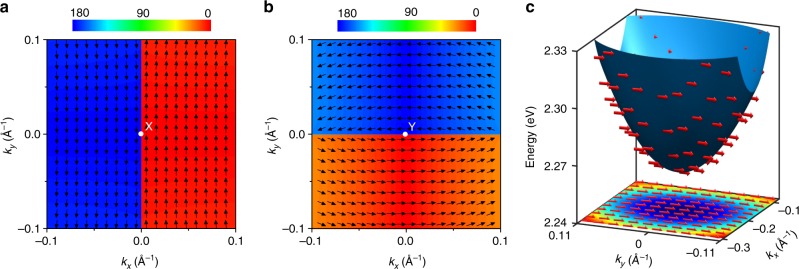
Spin texture of BiInO_3_. **a, b** Spin configurations around the high-symmetry k points: X point **a** and Y point **b**. The spin textures are plotted in the *k*_*z*_ = 0 plane for the lowest energy conduction bands. The wave vector k is referenced to the X point (**a**) and Y point (**b**), where it is assumed to be zero. The color map reflects the polar angle (in degrees) with respect to the *y*-axis (**a**) and *x*-axis (**b**). **c** 3D diagram and 2D projection of band structure and spin texture around the CBM. The arrows indicate the spin direction. The color map shows the energy profile. The wave vector is referenced to the X point where is it assumed to be zero

Next, we explore the spin texture around the X and Y points. According to Table 1, the space group No. 33 for BiInO_3_ supports the PST with the uniform spin orientation along the *y* (*x*) axis around the X (Y) point. This is exactly what we find from our DFT results. Figure 5a shows the calculated spin texture around the X point for the conduction band, which has the lowest energy. We see a unidirectional spin configuration for positive and negative values of *k*_*x*_ (referred to the origin being at the X point), which is consistent with the effective SOC field ${\mathbf{\Omega }}_{{\mathrm{PST}}} = \lambda (0,k_x,0)$ and the PST in Fig. 1. As expected, the spin orientation changes abruptly at *k*_*x*_ = 0, where the *s*_*y*_ component of the spin is reversed. We note that there is another band (with the opposite $\bar M_y$ eigenvalue if moving along the X–Γ line), which has higher energy (except the X–S line where it has the same energy) for which the spin has opposite orientation.

It is remarkable that the PST covers a substantial part of the Brillouin zone. The range of *k*_*x*_ and *k*_*y*_ values in Fig. 5a spans 0.2 Å^−1^ around the X point. For comparison, the *x* component of the reciprocal wave vector is *π*/*a* *=* 0.528 Å^−1^ (distance from the to X to Γ point). In fact, the nearly uniform spin structure persists even at larger distances and covers the CBM, which is located at about 0.19 Å^−1^ from the X point along the X–Γ line. Figure 5c shows the band dispersion and the spin structure around the CBM. It is evident that the spin maintains nearly unidirectional texture along the *y*-direction, which is reminiscent to that at the X point. Our calculations predict that in the range of *k*_*x*_ and *k*_*y*_ values spanning 0.2 Å^−1^ around the CBM (as in Fig. 5c), the largest deviation of the spin orientation from the *y*-axis is only 9.6°. This is due the CBM-forming band being well separated from the other two bands derived from the higher energy doublet (inset of Fig. 4b), so that the mixing between the doublets is minor. We note that the PST around the CBM is fully reversed when the wave vector **k** is changed to –**k**, due to time reversal symmetry.

The spin structure around the Y point (Fig. 5b) shows the similar trend, now with the spin being textured along the *x-*direction. The effective SOC field ${\mathbf{\Omega }}_{{\mathrm{PST}}} = \lambda (k_y,0,0)$ in this case leads to reversal of the *s*_*x*_ component when crossing the *k*_*y*_ *=* 0 line. There is a visible deviation from the unidirectional spin orientation when moving far away from the Y point. This stems from the reduced SOC splitting at the Y point (*Δ* ≈ 0.09 eV) as compared to that the X point (*Δ* ≈ 0.26 eV).

A ${\mathbf{k}} \cdot {\mathbf{p}}$ model. The spin textures around the high symmetry points can be further understood in terms of an effective ${\mathbf{k}} \cdot {\mathbf{p}}$ Hamiltonian, which we deduce from symmetry considerations. Here, we focus on the X point. In order to describe the four dispersing bands around the X point, additional sublattice degrees of freedom need to be included in the consideration. These are conventionally described by a set of Pauli matrices $\tau _j$ (*j* *=* *x*, *y*, *z*) operating in the sublattice space. The Hamiltonian around the X point is constructed by taking into account all symmetry operations at the X point, at which the symmetry generators are $\bar M_x$ and $\bar M_y$, and the time-reversal symmetry, which operator is $T = {\mathrm{i}}\sigma _yK$, where *K* is complex conjugation. We find that $\bar M_x$ and $\bar M_y$ can be represented as $\bar M_x = {\mathrm{i}}\tau _z\sigma _x$ and $\bar M_y = \tau _y\sigma _y$ (see Supplementary Note 2). Collecting all the terms up to linear order in **k**, which are invariant under these symmetry transformations, we obtain the ${\mathbf{k}} \cdot {\mathbf{p}}$ Hamiltonian:

5 $$\begin{array}{l}H = \delta \tau _y\sigma _y + \alpha k_x\tau _0\sigma _y + \beta k_y\tau _0\sigma _x + \gamma _1k_x\tau _y\sigma _0 + \\ {\kern 1pt} {\kern 1pt} {\kern 1pt} {\kern 1pt} {\kern 1pt} {\kern 1pt} {\kern 1pt} {\kern 1pt} {\kern 1pt} {\kern 1pt} {\kern 1pt} {\kern 1pt} {\kern 1pt} {\kern 1pt} {\kern 1pt} {\kern 1pt} \,{\kern 1pt} \gamma _2k_x\tau _x\sigma _x + \gamma _3k_x\tau _z\sigma _z + \gamma _4k_y\tau _x\sigma _y\,.\end{array}$$

Here, for simplicity we limit our consideration by the $(k_x,k_y)$ plane; *δ*, *α*, *β*, *γ*_*m*_ (*m* *=* 1–4) are independent parameters, $\sigma _0$ and $\tau _0$ are the 2 × 2 identity matrices, and direct products $\tau _i \otimes \sigma _j$ (*i*, *j* *=* 0, *x*, *y*, *z*) are implicitly assumed.

When *k* = 0 (i.e., at the X point), the $\delta \tau _y\sigma _y$ term in the Hamiltonian splits the state into two doublets distinguished by the eigenvalues of $\bar M_y = \pm 1$ and separated by $\Delta = 2\delta$. When *k* is not too large, the other terms in the Eq. (5) can be treated as perturbation. In the first order, the perturbation does not mix the doublets and the effective Hamiltonian within each of the doublets (labeled by indices $\pm$) can be written as

6 $$H^ \pm (k_x) = \pm \delta + \alpha ^ \pm k_x\sigma _y$$

where $\alpha ^ \pm = \sqrt {(\alpha \pm \gamma _1)^2 + (\gamma _2 \mp \gamma _3)^2}$ (see Supplementary Note 2). The corresponding eigenvalues are

7 $$\begin{array}{l}E_{}^ + (k_x) = \delta \pm \alpha ^ + k_x\\ E_{}^ - (k_x) = - \delta \pm \alpha ^ - k_x\end{array}$$

i.e., each doublet is split into two singlet states exhibiting linear dispersion away from the X point. This is consistent with our DFT results (inset in Fig. 4b). Fitting the DFT energy bands yields the following parameters: $\delta = - 0.13$ eV, $\alpha ^ + = 1.91$ eV Å, $\alpha ^ - = 1.51$ eV Å. Other parameters in the Hamiltonian of Eq. (5), can be found from the expectation values of the *y*-component of the spin, $s_y = \pm {\textstyle{1 \over 2}}(\alpha + \gamma _1)/\alpha ^ +$ and $s_y = \pm {\textstyle{1 \over 2}}(\alpha - \gamma _1)/\alpha ^ -$, for the doublet (+) and doublet (–), respectively. Using the DFT results for $s_y$, we obtain $\alpha = - 0.18$ eV Å, $\gamma _1 = - 1.42$ eV Å, $\gamma _2 = 0.95$ eV Å, and $\gamma _3 = 0.09$ eV Å.

The effective Hamiltonian of Eq. (6) imposes the effective SOC field pointing along the *y-*direction, i.e., ${\mathbf{\Omega }}_{{\mathrm{PST}}} = \lambda (0,k_x,0)$, where $\lambda = \alpha ^ \pm$, which produces the PST (Fig. 1c). Importantly, this form of the PST Hamiltonian appears as the result of the crystal symmetry rather than matching the Rashba and Dresselhaus constants. For the lowest energy band, $\lambda = \alpha ^ +$ and the spin is parallel (antiparallel) to the *y*-direction for positive (negative) *k*_*x*_. This is in agreement with the spin structure in Fig. 5a obtained from our DFT calculations.

Including first-order perturbation corrections to the wave function mixes states between the doublets, resulting in nonvanishing components of *s*_*x*_ and *s*_*z*_ and thus deviation from the PST. Our detailed analysis (see Supplementary Note 3) shows that within this approximation the $s_y$ component of the spin remains constant (Supplementary Eq. 21), whereas the $s_x$ component varies as $s_x = qk_y/\Delta$ (for the lowest conduction band), where *q* is a SOC constant (Supplementary Eq. 22). It is evident from this result that, first, $s_x = 0$ at $k_y = 0$, and hence the spin orientation remains collinear to the *y*-axis at the CBM ($k_x = 0.19$ Å^−1^, $k_y = 0$) as at the X point, and, second, when going away from the CBM along $k_y$ the $s_x$ value changes linear with $k_y$. Nonzero $s_x$ produces deviation from PST, but this deviation remains small over a broad area around the CBM due to the large splitting *Δ*. This is evident from Supplementary Fig. 3, which also reveals excellent agreement between the perturbation theory and explicit DFT calculation. This approach also allows us to obtain the remaining SOC constants in Hamiltonian of Eq. (5), $\beta = - 0.139$ eV Å and $\gamma _4 = 0.191$ eV Å, as detailed in Supplementary Note 3.

We would like to note that for the compounds considered in our work, any order terms in *k* in the Hamiltonian preserve PST in zero-order perturbation theory. Only in the first order of perturbation theory for the wave function a deviation from PST occurs with the dominant contribution resulting from linear in *k* terms. However, due to this contribution occurring as a perturbation, deviation from the PST remains small over a large area of the Brillouin zone.

## Discussion

The obtained value of the SOC parameter *λ* = 1.91 eV Å is three orders of magnitude larger than the values known for the semiconductor quantum-well structures (1–5 meV Å)^15–18^. It is also larger than the values predicted for other ferroelectric oxides, e.g., $\lambda _{\mathrm{R}} =$ 0.74 eV Å for BiAlO_3_ (*P*4*mm* space group)^26^ and $\lambda _{\mathrm{D}} =$0.58 eV Å for HfO_2_ (*Pca*2_1_ space group)^27^, and comparable to the value of $\lambda _{\mathrm{R}} =$3.85 eV Å observed in BiTeI^28^. The associated band splittings are sufficient to support room temperature functionalities. For example, the lowest excited state at ${\mathbf{k}} \approx (0.64{\mathrm{\pi }},0,0)$corresponding to the CBM lies about 0.29 eV above the CBM.

Electron motion in the PST state forms persistent spin helix (PSH)—the spatially periodic mode of spin polarization with the wave length of $l_{{\mathrm{PSH}}} = \frac{{{\mathrm{\pi }}\hbar ^2}}{{m\lambda }}$^13^. We estimate the effective mass *m* in BiInO_3_ by fitting the band dispersion around the CBM, which leads to *m* = 0.61*m*_0_, where *m*_0_ is the free electron mass. The resulting wave length is about 2 nm. This value is three orders of magnitude smaller than $l_{{\mathrm{PSH}}} \sim 5-10\,{\mathrm{\mu m}}$ observed in semiconductor heterostructures^16^.

It is conceivable (though challenging) to form and map a PSH state in BiInO_3_ in spirit of experiments by Walser et al.^16^. BiInO_3_ is a wide band gap semiconductor, and in order to observe this property an electron doping is required. Since Bi is isovalent to In, In_2_O_3_ may be considered as a comparative compound. It is known that oxygen vacancies naturally form in In_2_O_3_ producing *n*-type conductivity which can be varied over a broad range of magnitudes by changing growth conditions (mainly oxygen pressure)^29^. We expect, therefore, that a similar approach could be employed to produce electron doping in BiInO_3_. Due to CBM in BiInO_3_ maintaining PST, a PSH state will be formed if electrons are optically injected into the conduction band of BiInO_3_. Mapping the formation and evolution of PSH in BiInO_3_ could possibly be performed using near-field scanning Kerr microscopy, which showed a possibility to resolve features down to tens-nm scale with sub-ns time resolution^30^. In addition, the electron-doped BiInO_3_ can be used to explore the current induced spin polarization (known as the Edelstein effect^7^) and associated spin-orbit torques^31^, which are expected to be large due to the large SOC.

We also envision a possibility to observe a Hall effect qualitatively similar to the valley Hall effect recently discovered in transition metal dichalcogenides (TMD)^32^. In BiInO_3_ the two states with **k** and **-k** at the CBM with opposite spin orientation are related by time reversal symmetry transformation and thus have opposite sign of the Berry curvature. If an imbalance in electron population between these two states is created by polarized optical excitation (similar to that done in TMDs), a charge Hall current can be measured that reverses sign with polarization of the exciting light.

Another implication is a possibility to use a PST material as a barrier in tunnel junctions. It has been predicted that the Rashba and Dresselhaus SOC in a tunnel barrier can produce tunneling anomalous and spin Hall effects^33,34^. Using a PST material as a tunnel barrier allows producing a perfect anisotropy in the Hall response. For example, if the current flows in the *z-*direction across a PST barrier with the SOC given by Eq. (6), the tunneling Hall response will be zero in the *y*-direction and nonzero in the *x*-direction. Moreover, the anomalous Hall conductivity is expected to strongly depend on the magnetization orientation in the *x–y* plane and vanish for magnetization pointing along the *x*-direction. The large value of *λ* = 1.91 eV Å is expected to produce sizable effects, which can be detected experimentally. In addition, the reversible spin texture of ferroelectric SOC oxide materials^35,36^ will support the tunneling Hall effects to be reversible by an applied electric field through switching of ferroelectric polarization^27^.

Apart from BiInO_3_, there are a number of other potential candidates which are expected to maintain a PST. Among them are BiInS_3_ (*Pna*2_1_ structure, space group No. 33) and LiTeO_3_ (*Pnn*2 structure, space group No. 34). Both have a PST around the high symmetry X and Y points (see Supplementary Note 4). BiInS_3_ has a lower calculated band gap (about 1.13 eV), but a CBM is located at the Γ point, which does not support the PST. On the other hand, LiTeO_3_ (calculated band gap is about 2 eV) has a CBM close to the X point similar to BiInO_3_.

Overall, we have demonstrated that the PST is imposed by symmetry in a class of orthorhombic nonsymmorphic bulk materials, such as BiInO_3_. The PST is a robust intrinsic property of these materials, which eliminates the stringent condition of equal Rashba and Dresselhaus SOC for realizing the persistent spin helix. The electronic and spin properties of the PST materials are derived from the nontrivial interplay between spin–orbit coupling and glide reflection symmetries, and in this regard place them among interesting quantum materials which have recently received a lot of attention. We hope, therefore, that our theoretical predictions will stimulate experimental efforts in the exploration of these materials, which functional properties may be useful for device applications.

## Methods

### DFT calculations

DFT calculations are performed using a plane-wave pseudopotential method implemented in Quantum-ESPRESSO^37^. In the calculations, we use the lattice constants and atomic positions of bulk materials, which are given in Supplementary Note 4. The exchange-correlation functional is treated within the generalized gradient approximation^38^. We use energy cutoff of 544 eV for the plane wave expansion and 10 × 10 × 8 *k*-point grid for Brillouin zone integrations. The electric polarization is computed using the Berry phase method^39^. SOC is included in the calculations using the fully relativistic ultrasoft pseudopotentials^40^. The expectation values of the spin operators $s_i = {\textstyle{1 \over 2}}\left\langle {\psi _{\mathbf{k}}} \right|\sigma _i\left| {\psi _{\mathbf{k}}} \right\rangle$ (*i* = *x*, *y*, *z*) are obtained directly from the noncollinear spin DFT calculations. The atomic structures are produced using VESTA software^41^.

### Data availability

The data that support the findings of this study are available from the authors upon request.

## Electronic supplementary material

Supplementary Information
