# Supplementary material for: Persistent spin texture enforced by symmetry
Source: Nat Commun. 2018 Jul 17;9:2763. doi: 10.1038/s41467-018-05137-0 (PMC6050308; doi:10.1038/s41467-018-05137-0)
Supplement: Supplementary file 1 — Supplementary Information [file 41467_2018_5137_MOESM1_ESM.pdf]

Supplementary Information

Persistent spin texture enforced by symmetry

Tao *et al.*

Supplementary Note 1.

Projected density of states, hourglass band dispersion, and a nodal line in $BiInO_3$

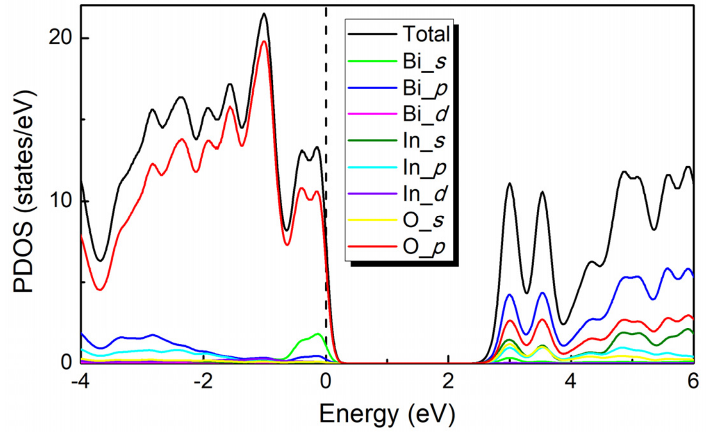

Supplementary Figure 1. Projected density of states. Projected density of states (PDOS) for bulk $BiInO_3$ without SOC.

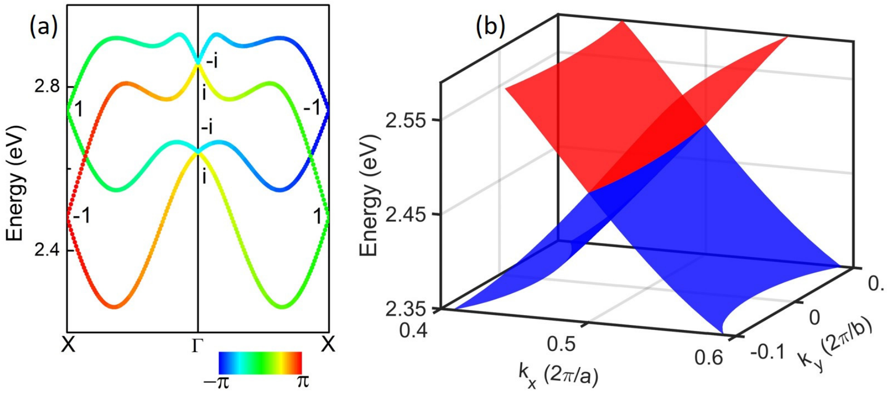

Supplementary Figure 2. Hourglass-shaped band dispersion and node line. (a) Lowest energy conduction bands along the Γ-X symmetry line. The color map quantifies the phase of the eigenvalues of $\bar{M}_y$. The numbers represent the eigenvalues at the time-reversal-invariant momenta, i.e. Γ and X. (b) Three-dimensional band dispersion for the lowest two conduction bands around the X point.

As discussed in the main text, along the Γ-X line, where $\mathbf{k}=(k_x,0,0)$, $\mathbf{k}$ is invariant under $\bar{M}_y$ operation. Note that transformation rule for wave vector $\mathbf{k}$ (k_x, k_y, k_z) under the glide reflection is the same as that under the mirror reflection. Each Bloch state $\psi_{\mathbf{k}}$ can be labeled using the eigenvalues of $\bar{M}_y$: $\bar{M}_y\left|\psi_{\mathbf{k}}^{\pm}\right\rangle=\pm \mathrm{i}e^{-\mathrm{i}\frac{k_x}{2}}\left|\psi_{\mathbf{k}}^{\pm}\right\rangle$. In addition, along this line, $\tilde{\Theta}^2=T^2\bar{M}_x^2=e^{-\mathrm{i}k_y-\mathrm{i}k_z}=1$, and thus $\tilde{\Theta}$ cannot result in the Kramers-like double degeneracy. T enforces the band degeneracy at the time-reversal-invariant momenta (TRIMs) $\mathbf{k}$, which satisfy $\mathbf{k}=-\mathbf{k}+\mathbf{G}$ with $\mathbf{G}$ being the reciprocal lattice vector. At the TRIMs, the Kramers pair has the complex-conjugate eigenvalues of $\bar{M}_y$, namely (i, – i) and (–i, i) at the Γ point. In contrast, the eigenvalues for Kramers pair are (1, 1) and (–1, –1) at the X point. The eigenvalue of $\bar{M}_y$ must evolve continuously from i to 1 or from -i to –1, resulting in an hourglass-shaped band

dispersion (Supplementary Fig. 2a).[1,2] The band crossing occurs between the Γ and X point, which is protected by the $\bar{M}_y$ symmetry and robust against the SOC. This is seen from the following consideration. Assume that there exists perturbation H' in the system. Since $\bar{M}_y\left|\psi_{\mathbf{k}}^{\pm}\right\rangle = \pm \mathrm{i}\mathrm{e}^{-\mathrm{i}\frac{k_x}{2}}\left|\psi_{\mathbf{k}}^{\pm}\right\rangle$, the matrix element of H' is given by $\left\langle\psi_{\mathbf{k}}^{+}\left|H'\right|\psi_{\mathbf{k}}^{-}\right\rangle = \left\langle\psi_{\mathbf{k}}^{+}\left|\bar{M}_y^{-1}H'\bar{M}_y\right|\psi_{\mathbf{k}}^{-}\right\rangle = -\left\langle\psi_{\mathbf{k}}^{+}\left|H'\right|\psi_{\mathbf{k}}^{-}\right\rangle$, which implies $\left\langle\psi_{\mathbf{k}}^{+}\left|H'\right|\psi_{\mathbf{k}}^{-}\right\rangle = 0$. Thus, any perturbation that does not break the $\bar{M}_y$ symmetry cannot induce hybridization between the states with different $\bar{M}_y$ eigenvalues. The crossing point is therefore protected by the $\bar{M}_y$ symmetry. On the other hand, while the bands are split along the Γ-X line, they are double degenerate along the X-S line. Therefore, a node line along $\mathbf{k} = (\pm\pi, k_y, 0)$ is naturally formed (Supplementary Fig. 2b).

Supplementary Note 2.

A model Hamiltonian

Here, we derive the $\mathbf{k}\cdot\mathbf{p}$ effective Hamiltonian for $BiInO_3$ around the X point and determine the eigenvalues and eigenstates using the perturbation theory. The $\mathbf{k}\cdot\mathbf{p}$ Hamiltonian around the X point can be constructed by considering all the symmetry operations at the X point[3], at which the symmetry generators are $\bar{M}_x$ and $\bar{M}_y$. As shown in the main text, there are two conjugated doublets at the X point, $(\psi_{\mathrm{X}}^{+}, \Theta\psi_{\mathrm{X}}^{+})$ or $(\psi_{\mathrm{X}}^{-}, \Theta\psi_{\mathrm{X}}^{-})$, which are distinguished by the $\bar{M}_y$ eigenvalues and $\Theta \equiv T\bar{M}_y$. To describe these four states, in addition to spin, sublattice degrees of freedom need to be included in the consideration, which are conventionally described by a set of Pauli matrices τ_j ($j = x, y, z$). The time-reversal symmetry T is represented as $T = \mathrm{i}\sigma_y K$, where K is complex conjugation. The $\bar{M}_y$ operator in the spin space is described by $\mathrm{i}\sigma_y$. In order to determine its effect on the pseudospin $\boldsymbol{\tau}$, we take into account the fact that $\bar{M}_y^2 = 1$ at the X point and that $[T, \bar{M}_y] = 0$. This leads to $\bar{M}_y = \tau_y\sigma_y$. In order to determine $\bar{M}_x$, we calculate the eigenvalues of $\bar{M}_x$ for the doublets $(\psi_{\mathrm{X}}^{+}, \Theta\psi_{\mathrm{X}}^{+})$ or $(\psi_{\mathrm{X}}^{-}, \Theta\psi_{\mathrm{X}}^{-})$. Since $\bar{M}_x^2 = -\mathrm{e}^{-\mathrm{i}k_y} = -1$ at the X point, we have $\bar{M}_x\left|\psi_{\mathrm{X}}^{\pm}\right\rangle = \pm\mathrm{i}\left|\psi_{\mathrm{X}}^{\pm}\right\rangle$. Now we calculate the eigenvalue of $\bar{M}_x$ for the conjugated state $\Theta\left|\psi_{\mathrm{X}}^{\pm}\right\rangle$. The commutation relation between $\bar{M}_x$ and $\bar{M}_y$ can be derived from the following successive symmetry operations. In real space, we obtain

$$\left.\begin{array}{l}(x,y,z)\xrightarrow{\bar{M}_x}\left(-x+\frac{1}{2},y+\frac{1}{2},z+\frac{1}{2}\right)\xrightarrow{\bar{M}_y}\left(-x+1,-y,z+\frac{1}{2}\right)\\(x,y,z)\xrightarrow{\bar{M}_y}\left(x+\frac{1}{2},-y+\frac{1}{2},z\right)\xrightarrow{\bar{M}_x}\left(-x,-y+1,z+\frac{1}{2}\right)\end{array}\right\}\Rightarrow \bar{M}_x\bar{M}_y = \mathrm{e}^{-\mathrm{i}k_x+\mathrm{i}k_y}\bar{M}_y\bar{M}_x. \quad (1)$$

In the spin space, $\bar{M}_x\bar{M}_y = -\bar{M}_y\bar{M}_x$ due to $\bar{M}_x = \mathrm{i}\sigma_x$ and $\bar{M}_y = \mathrm{i}\sigma_y$. Combining the real space and spin space, we obtain $\bar{M}_x\bar{M}_y = -\mathrm{e}^{-\mathrm{i}k_x+\mathrm{i}k_y}\bar{M}_y\bar{M}_x$. At the X point ($k_x = \pi$, $k_y = 0$), we then have $\left[\bar{M}_x, \bar{M}_y\right] = 0$. Thus, $\bar{M}_x\Theta\left|\psi_{\mathrm{X}}^{\pm}\right\rangle = \Theta\bar{M}_x\left|\psi_{\mathrm{X}}^{\pm}\right\rangle = \mp\mathrm{i}\Theta\left|\psi_{\mathrm{X}}^{\pm}\right\rangle$, implying that the eigenvalues for the doublets $(\psi_{\mathrm{X}}^{+}, \Theta\psi_{\mathrm{X}}^{+})$ or $(\psi_{\mathrm{X}}^{-}, \Theta\psi_{\mathrm{X}}^{-})$ have opposite sign. To satisfy all these conditions $\bar{M}_x$ can be chosen as $\bar{M}_x = \mathrm{i}\tau_z\sigma_x$. From the expressions for T, $\bar{M}_x$, and $\bar{M}_y$ given above, it is easy to see that $\left[T, \bar{M}_x\right] = 0$ and $\left[T, \bar{M}_y\right] = 0$ in the spin space. Since T does not change the real-space coordinates, the commutation relations $\left[T, \bar{M}_x\right] = 0$ and $\left[T, \bar{M}_y\right] = 0$ also hold in the real space. The corresponding transformations for $\mathbf{k}$, $\boldsymbol{\sigma}$ and $\boldsymbol{\tau}$ are given in Supplementary Table 1.

Supplementary Table 1. Transformation rules for wave vector **k**, and spin (σ) and sublattice (τ) Pauli matrices under the C_{2v} point-group symmetry operations at the X (π, 0, 0) point in the Brillouin zone of $BiInO_3$. The wave vector **k** is referenced with respect to the high symmetry point where it is assumed to be zero. K denotes complex conjugation.

**Symmetry**	(k_x, k_y, k_z)	$(\sigma_x, \sigma_y, \sigma_z)$	(τ_x, τ_y, τ_z)
$T = i\sigma_y K$	$(-k_x, -k_y, -k_z)$	$(-\sigma_x, -\sigma_y, -\sigma_z)$	$(\tau_x, -\tau_y, \tau_z)$
$\bar{M}_x = i\tau_z\sigma_x$	$(-k_x, k_y, k_z)$	$(\sigma_x, -\sigma_y, -\sigma_z)$	$(-\tau_x, -\tau_y, \tau_z)$
$\bar{M}_y = \tau_y\sigma_y$	$(k_x, -k_y, k_z)$	$(-\sigma_x, \sigma_y, -\sigma_z)$	$(-\tau_x, \tau_y, -\tau_z)$
**Commutation**	$\left[T, \bar{M}_x\right] = 0$, $\left[T, \bar{M}_y\right] = 0$, $\left[\bar{M}_x, \bar{M}_y\right] = 0$, $\bar{M}_x^2 = -1$, $\bar{M}_y^2 = 1$		

We limit our consideration by dispersion in the (k_x, k_y) plane. Collecting all the terms up to linear order in **k** (the quadric and cubic in k terms are listed in Supplementary Table 3 of Supplementary Note 6), which are invariant under these symmetry transformations, we obtain the $\mathbf{k}\cdot\mathbf{p}$ Hamiltonian as follows:

$$H = \delta\tau_y\sigma_y + \alpha k_x\tau_0\sigma_y + \beta k_y\tau_0\sigma_x + \gamma_1 k_x\tau_y\sigma_0 + \gamma_2 k_x\tau_x\sigma_x + \gamma_3 k_x\tau_z\sigma_z + \gamma_4 k_y\tau_x\sigma_y \ , \tag{2}$$

where **k** is referred to the X point. We split the Hamiltonian of Eq. (2) into $H = H_0 + H'$, where

$$H_0 = \delta\tau_y\sigma_y \tag{3}$$

is k independent term and

$$H' = \alpha k_x\tau_0\sigma_y + \beta k_y\tau_0\sigma_x + \gamma_1 k_x\tau_y\sigma_0 + \gamma_2 k_x\tau_x\sigma_x + \gamma_3 k_x\tau_z\sigma_z + \gamma_4 k_y\tau_x\sigma_y \tag{4}$$

is k dependent term. H' can be treated as perturbation when k is small (measured from the X point). The basis set can be constructed as the direct product of the eigenstates for pseudospin τ and spin σ. It is convenient to choose basis functions to be the eigenstates of τ_y and σ_y. We use χ and ξ to denote the spin eigenstates for τ_y and σ_y, respectively, so that $\chi_+ = \frac{1}{\sqrt{2}}\begin{bmatrix}1\\ \mathrm{i}\end{bmatrix}$, $\chi_- = \frac{1}{\sqrt{2}}\begin{bmatrix}\mathrm{i}\\ 1\end{bmatrix}$ and $\xi_+ = \frac{1}{\sqrt{2}}\begin{bmatrix}1\\ \mathrm{i}\end{bmatrix}$, $\xi_- = \frac{1}{\sqrt{2}}\begin{bmatrix}\mathrm{i}\\ 1\end{bmatrix}$. Then the basis set is as follows

$$\begin{cases}\phi_1 = \chi_+\xi_+ \\ \phi_2 = \chi_-\xi_- \\ \phi_3 = \chi_+\xi_- \\ \phi_4 = \chi_-\xi_+\end{cases} . \tag{5}$$

Hamiltonian H_0 in the basis set is diagonal, so that $H_0 = \mathrm{diag}\left[\delta, \delta, -\delta, -\delta\right]$, and its eigenvalues are

$$\begin{cases}E_{1,2}^{(0)} = \delta \\ E_{3,4}^{(0)} = -\delta\end{cases} . \tag{6}$$

In the basis of ϕ_j (j = 1–4), the matrix elements of the spin operators σ_j ($j = x, y, z$) can be expressed as

$$\sigma_x = \begin{bmatrix} 0 & 0 & 1 & 0 \\ 0 & 0 & 0 & 1 \\ 1 & 0 & 0 & 0 \\ 0 & 1 & 0 & 0 \end{bmatrix},\ \sigma_y = \begin{bmatrix} 1 & 0 & 0 & 0 \\ 0 & -1 & 0 & 0 \\ 0 & 0 & -1 & 0 \\ 0 & 0 & 0 & 1 \end{bmatrix},\ \sigma_z = \begin{bmatrix} 0 & 0 & i & 0 \\ 0 & 0 & 0 & -i \\ -i & 0 & 0 & 0 \\ 0 & i & 0 & 0 \end{bmatrix}. \tag{7}$$

We see that the eigenstates of the Hamiltonian H_0 diagonalize the σ_y matrix (i.e. are eigenstates of σ_y), which is consistent with the symmetry arguments of the main text.

Next, we follow the standard degenerate perturbation theory to determine the eigenvalues and eigenstates. In the basis of ϕ_j (j = 1–4), H' can be expressed as

$$H' = \begin{bmatrix} \alpha k_x + \gamma_1 k_x & \gamma_2 k_x - \gamma_3 k_x & \beta k_y & \gamma_4 k_y \\ \gamma_2 k_x - \gamma_3 k_x & -\alpha k_x - \gamma_1 k_x & -\gamma_4 k_y & \beta k_y \\ \beta k_y & -\gamma_4 k_y & -\alpha k_x + \gamma_1 k_x & \gamma_2 k_x + \gamma_3 k_x \\ \gamma_4 k_y & \beta k_y & \gamma_2 k_x + \gamma_3 k_x & \alpha k_x - \gamma_1 k_x \end{bmatrix}. \tag{8}$$

The first order correction to the energy $E^{(1)}$ is

$$\begin{cases} E_1^{(1)} = -\alpha^+ k_x,\ E_2^{(1)} = \alpha^+ k_x \\ E_3^{(1)} = -\alpha^- k_x,\ E_4^{(1)} = \alpha^- k_x \end{cases}, \tag{9}$$

where

$$\alpha^{\pm} = \sqrt{(\alpha \pm \gamma_1)^2 + (\gamma_2 \mp \gamma_3)^2}, \tag{10}$$

and the eigenstates $\psi^{(0)}$ are

$$\begin{cases} \psi_1^{(0)} = \dfrac{1}{\sqrt{c_1^2+1}}(\phi_1 + c_1\phi_2),\ \psi_2^{(0)} = \dfrac{1}{\sqrt{c_1^2+1}}(-c_1\phi_1 + \phi_2) \\ \psi_3^{(0)} = \dfrac{1}{\sqrt{c_2^2+1}}(\phi_3 - c_2\phi_4),\ \psi_4^{(0)} = \dfrac{1}{\sqrt{c_2^2+1}}(c_2\phi_3 + \phi_4) \end{cases}, \tag{11}$$

where the parameters c_j (j = 1–2) are defined as

$$\begin{cases} c_1 = \dfrac{\gamma_2 - \gamma_3}{\alpha + \gamma_1 - \alpha^+} \\ c_2 = \dfrac{\gamma_2 + \gamma_3}{\alpha - \gamma_1 + \alpha^-} \end{cases}. \tag{12}$$

Within the basis set of functions (Supplementary Eq. (11)) the matrix elements of σ_j ($j = x, y, z$) are given by

$$\sigma_x = \frac{1}{\sqrt{(c_1^2+1)(c_2^2+1)}}\begin{bmatrix} 0 & 0 & 1-c_1c_2 & c_1+c_2 \\ 0 & 0 & -c_1-c_2 & 1-c_1c_2 \\ 1-c_1c_2 & -c_1-c_2 & 0 & 0 \\ c_1+c_2 & 1-c_1c_2 & 0 & 0 \end{bmatrix}$$

$$\sigma_y = \begin{bmatrix} \frac{1-c_1^2}{c_1^2+1} & 0 & 0 & 0 \\ 0 & \frac{c_1^2-1}{c_1^2+1} & 0 & 0 \\ 0 & 0 & \frac{c_2^2-1}{c_2^2+1} & 0 \\ 0 & 0 & 0 & \frac{1-c_2^2}{c_2^2+1} \end{bmatrix}$$

$$\sigma_z = \frac{\mathrm{i}}{\sqrt{(c_1^2+1)(c_2^2+1)}}\begin{bmatrix} 0 & 0 & 1+c_1c_2 & c_2-c_1 \\ 0 & 0 & c_2-c_1 & -1-c_1c_2 \\ -1-c_1c_2 & c_1-c_2 & 0 & 0 \\ c_1-c_2 & 1+c_1c_2 & 0 & 0 \end{bmatrix}. \tag{13}$$

We see that in agreement with our general consideration given in the main text, the matrix elements of the spin operators σ_x and σ_z within the two doublets are equal to zero (two 2×2 block diagonal matrices). On the other hand, we also see that $\psi_j^{(0)}$ (j = 1–4) are the eigenstates of σ_y and have eigenvalues of opposite sign for the two states within either doublet. This allows us writing an effective Hamiltonian up to the first order in perturbation theory within each of the two doublets (labelled by indices $\pm$) in the form of in Eq. (6) of the main text, i.e.

$$H^{\pm}(k_x) = \pm\delta + \alpha^{\pm}k_x\sigma_y\,. \tag{14}$$

We note that, the expectation values of $s_y = \frac{1}{2}\langle\sigma_y\rangle$ are not equal to $\pm\frac{1}{2}$ but in general deviate from these values.[4] Using Supplementary Eqs. (12) and (13), we find

$$s_y = \pm\frac{1}{2}\frac{\alpha+\gamma_1}{\alpha^{+}} \tag{15}$$

and

$$s_y = \pm\frac{1}{2}\frac{\alpha-\gamma_1}{\alpha^{-}} \tag{16}$$

for doublet (+) and doublet (–), respectively.

These relationships can be used to find all the parameters in the model Hamiltonian Supplementary Eq. (2). The values of δ , α^{+}, and α^{-} are obtained by fitting the DFT band dispersions with Supplementary Eqs. (6) and (9). Then using the DFT calculated values s_y and Supplementary Eqs. (15) and (16) we can find parameters α and γ_1, using these values and Supplementary Eqs. (10), obtain γ_2 and γ_3. The results are as follows: $\delta = -0.13$ eV, $\alpha^{+} = 1.91$ eV Å, $\alpha^{-} = 1.51$ eV Å, $\alpha = -0.18$ eV Å, $\gamma_1 = -1.42$ eV Å, $\gamma_2 = 0.95$ eV Å and $\gamma_3 = 0.09$ eV Å.

Supplementary Note 3.

Higher order corrections and deviations from PST

Second-order corrections to the energy $E^{(2)}$ are given by

$$\begin{cases} E_1^{(2)} = E_2^{(2)} = \frac{t}{\Delta} k_y^2 \\ E_3^{(2)} = E_4^{(2)} = -\frac{t}{\Delta} k_y^2 \end{cases}, \tag{17}$$

where $\Delta = 2\delta$ is the zero-order energy splitting and t is defined as follows

$$\begin{cases} t = |C_1|^2 + |C_2|^2 \\ C_1 = \frac{\beta - c_1\gamma_4 - c_2\gamma_4 - c_1c_2\beta}{\sqrt{(c_1^2+1)(c_2^2+1)}} \\ C_2 = \frac{c_2\beta - c_1c_2\gamma_4 + \gamma_4 + c_1\beta}{\sqrt{(c_1^2+1)(c_2^2+1)}} \end{cases}. \tag{18}$$

Thus, up to the second-order perturbation, the eigenvalues $E_j = E_j^{(0)} + E_j^{(1)} + E_j^{(2)}$ $(j = 1-4)$ are given by

$$\begin{cases} E_1 = \delta - \alpha^+ k_x + \frac{t}{\Delta} k_y^2 \\ E_2 = \delta + \alpha^+ k_x + \frac{t}{\Delta} k_y^2 \\ E_3 = -\delta - \alpha^- k_x - \frac{t}{\Delta} k_y^2 \\ E_4 = -\delta + \alpha^- k_x - \frac{t}{\Delta} k_y^2 \end{cases}. \tag{19}$$

First-order corrections $\psi_j^{(1)}$ to the eigenstates $\psi_j^{(0)}$ Supplementary (11) produce first-order eigenstates $\psi_j = \psi_j^{(0)} + \psi_j^{(1)}$ $(j = 1-4)$ as follows:

$$\begin{cases} \psi_1 = \psi_1^{(0)} + \frac{C_1 k_y}{\Delta}\psi_3^{(0)} + \frac{C_2 k_y}{\Delta}\psi_4^{(0)} \\ \psi_2 = \psi_2^{(0)} - \frac{C_2 k_y}{\Delta}\psi_3^{(0)} + \frac{C_1 k_y}{\Delta}\psi_4^{(0)} \\ \psi_3 = \psi_3^{(0)} - \frac{C_1 k_y}{\Delta}\psi_1^{(0)} + \frac{C_2 k_y}{\Delta}\psi_2^{(0)} \\ \psi_4 = \psi_4^{(0)} - \frac{C_2 k_y}{\Delta}\psi_1^{(0)} - \frac{C_1 k_y}{\Delta}\psi_2^{(0)} \end{cases}, \tag{20}$$

where C_1 and C_2 are given by Supplementary Eq. (18).

From Supplementary Eqs. (13) and (20), we see that the perturbed eigenstates are no longer the eigenstates of σ_y due to mixing between the doublets. Taking state ψ_1, which corresponds to the lowest conduction band in Supplementary Eq. (20), as an example, we find the expectation values of s_x and s_y up to the first order in perturbation theory:

$$\begin{cases} s_x = \dfrac{1}{2}\dfrac{\langle\psi_1|\sigma_x|\psi_1\rangle}{\langle\psi_1|\psi_1\rangle} = q\dfrac{k_y}{\Delta} \\ s_y = \dfrac{1}{2}\dfrac{\langle\psi_1|\sigma_y|\psi_1\rangle}{\langle\psi_1|\psi_1\rangle} = \dfrac{1-c_1^2}{2\left(c_1^2+1\right)} \end{cases}, \tag{21}$$

where

$$q = \frac{\left(1-c_1c_2\right)C_1 + \left(c_1+c_2\right)C_2}{\sqrt{\left(c_1^2+1\right)\left(c_2^2+1\right)}} \ . \tag{22}$$

Note that in Supplementary Eq. (21) we omitted the quadratic terms in k_y as they correspond to the higher-order perturbation. We see that within this approximation, the s_y component of the spin remains unchanged and constant, whereas the s_x component becomes nonzero and linear in k_y.

Supplementary Eqs. (19) and (21) can be used to obtain the two yet undermined constants in the model Hamiltonian i.e. Supplementary Eq. (2), i.e. β and γ_4. By fitting the DFT calculated band structure along the high-symmetry X-S direction ($k_x = \pi$, k_y, $k_z = 0$) we find parameter $t = 0.0559$ eV^2 Å^2 and by fitting the DFT calculated value of s_x as a function k_y we find parameter $q = -0.139$ eV Å. From t and q using Supplementary Eqs. (18) and (22), we obtain $C_1 = -0.161$ eV Å and $C_2 = 0.173$ eV Å. Substituting C_1 and C_2 into Supplementary Eq.(18), we finally find $\beta = -0.139$ eV Å and $\gamma_4 = 0.191$ eV Å.

Supplementary Fig. 3 shows results for the spin texture around the CBM calculated from first-principles (Supplementary Fig. 3a) and using our model Hamiltonian within the perturbation approach (Supplementary Fig. 3b). First, it is seen that over a very broad region around the CBM the spin magnitude and orientation is nearly uniform. Second, qualitative comparison between the DFT computed and modeled spin textures reveals excellent agreement. This is remains the case when the comparison is made quantitative. As is evident from Supplementary Fig. 3d, s_x increases linearly with k_y consistent with the model prediction. Supplementary Fig. 3e shows that s_y is nearly independent of k, which is again in line with the result of Supplementary Eq. (21). Supplementary Fig. 3c shows the calculated spin-orbit field around the CBM. As expected, the magnitude of the spin-orbit field (arrow length) scales linear with k_x (referenced to the X point) and weakly depends on k_y.

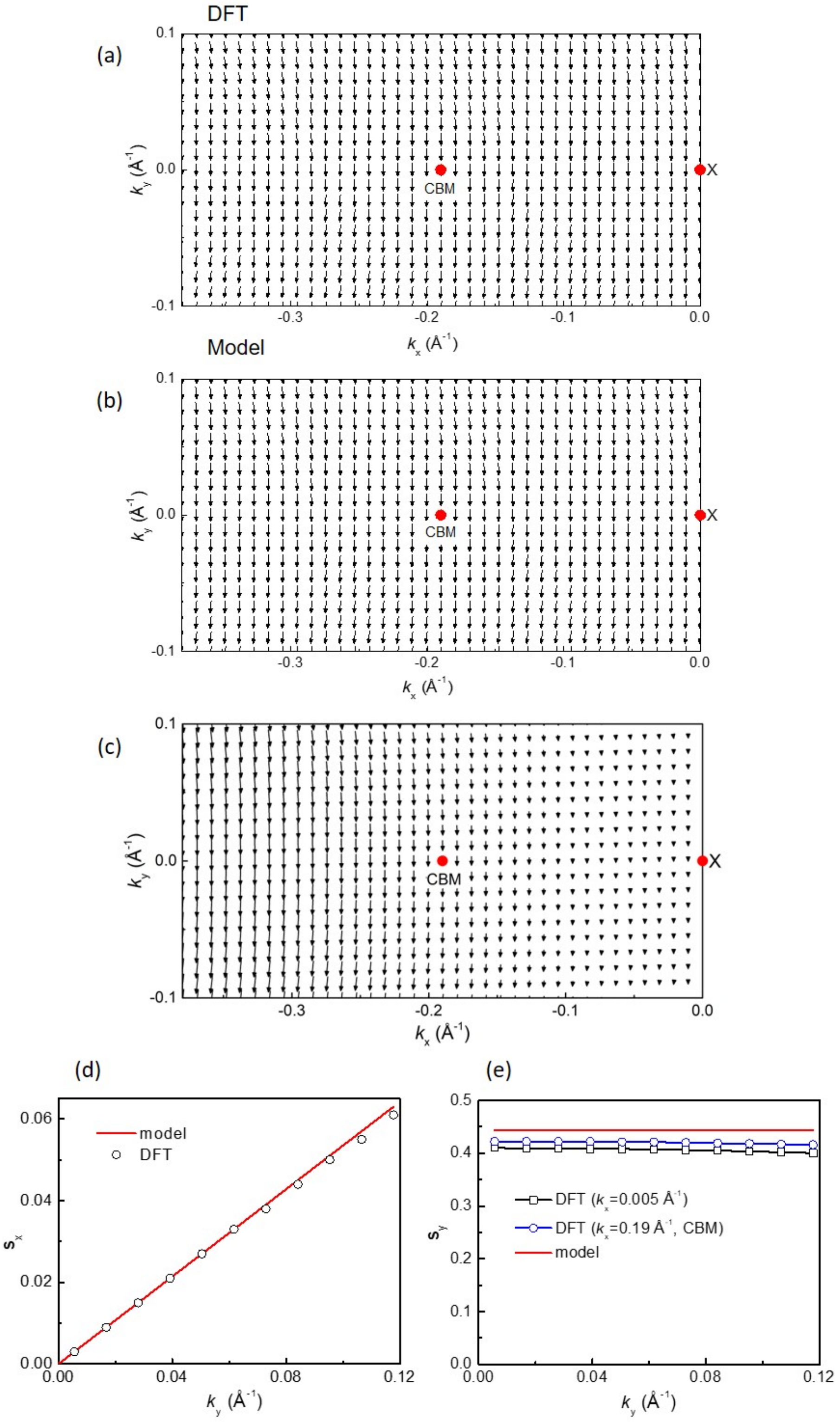

Supplementary Figure 3. Spin texture around the CBM. (a, b) Spin textures around the CBM in the $k_z = 0$ plane: DFT results (a) and model results (b) based on Eq. (21). k_x is measured from the X point. (c) Spin-orbit field around the CBM based on the effective Hamiltonian model. (d) Expectation value of the *x* component of the spin, s_x. The model results are represented by $s_x = qk_y / \Delta$, where $q / \Delta = 0.535$ Å and k_x = 0.19 Å^{-1} corresponding to CBM. (e) Expectation value of the *y* component of the spin, s_y. The model results are represented by Supplementary Eq. (21).

Supplementary Note 4.

Other PST compounds

Supplementary Fig. 4 shows the crystal structures of $BiInS_3$ and $LiTeO_3$. The orthorhombic *Pna*2_1 structure of $BiInS_3$ (space group No. 33) was proposed in experiment [5] and predicted by first-principles.[6] In our DFT calculations, we used the theoretically predicted lattice constants and atomic coordinates from Supplementary ref.6. The $LiTeO_3$ compound with *Pnn*2 (No. 34) structure was predicted by the Material Project,[7] but the experimental demonstration of this structural phase has not yet been reported. In this work, the lattice constants and atomic coordinates for bulk $LiTeO_3$ were obtained using full structural relaxation with the initial geometry adopted from Supplementary ref.7. Supplementary Table 2 summarizes the lattice constants, atomic positions, calculated polarizations and band gaps for all the three compounds considered in this work.

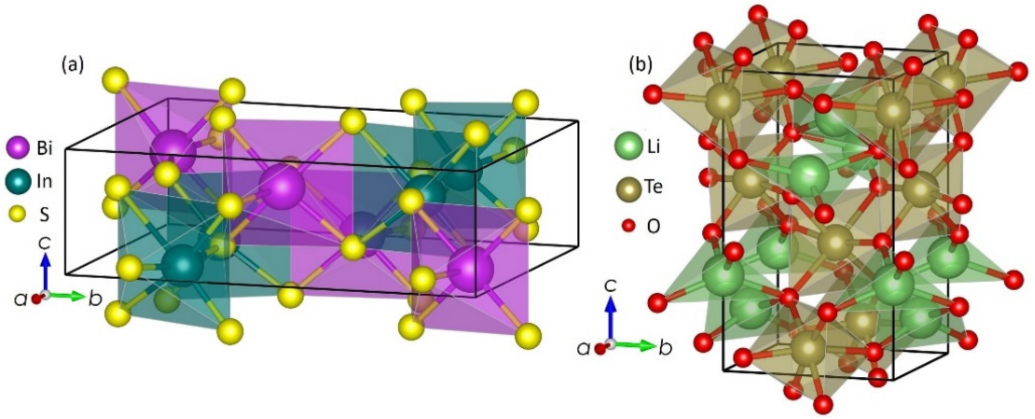

Supplementary Figure 4. **Crystal structure of bulk $BiInS_3$ and $LiTeO_3$.** Crystal structure of bulk (a) $BiInS_3$ in the *Pna*2_1 (No. 33) orthorhombic phase and (b) $LiTeO_3$ in the *Pnn*2 (No. 34) orthorhombic phase. Here (*a*, *b*, *c*) axis is concordant with the (*x*, *y*, *z*) axis.

All the three compounds have finite polarization due to broken inversion symmetry. For the orthorhombic crystal system with two perpendicular mirror reflections M_x and M_y (Fig. 1a in the main text), polarization along the *x* or *y* direction is forbidden by symmetry. The calculated polarization along the *z* direction is listed in Table S2. We see that polarizations of $BiInO_3$ and $LiTeO_3$ are similar and comparable to the polarization of conventional ferroelectric oxide $BaTiO_3$, whereas the polarization of $BiInS_3$ is smaller (but still sizeable).

Supplementary Table 2. Structural and electronic properties of $BiInO_3$, $BiInS_3$, and $LiTeO_3$ compounds: lattice constants, atomic positions, calculated polarizations P_z, band gaps, and energy splittings Δ_X (Δ_Y) between the two lowest conduction bands at the X (Y) point. The band gaps are calculated using GGA in presence of SOC.

Compound	Atom	Wyckoff position	*x*	*y*	*z*	P_z (μC/cm^2)	Band gap (eV)	Δ_X (eV)	Δ_Y (eV)
**$BiInO_3$**	Bi1	4a	0.05955	0.00880	0.77970	33.6	2.26	0.26	0.09
a = 5.955 Å	In1	4a	0.00260	0.50110	0.00000				
b = 5.602 Å	O1	4a	0.05400	0.38300	0.77300				
c = 8.386 Å	O2	4a	0.17100	0.21800	0.44700				
Ref. 8	O3	4a	0.34400	0.63000	0.53000				
**$BiInS_3$**	Bi1	4a	0.67016	0.44544	0.64635	11.4	1.13	0.03	0.05
a =10.060 Å	In1	4a	0.07064	0.73238	0.59238				
b =13.380 Å	S1	4a	0.39540	0.39764	0.66734				
c =3.940 Å	S2	4a	0.95118	0.38956	0.59942				
Ref. 6	S3	4a	0.82959	0.80091	0.65975				
**$LiTeO_3$**	Li1	2a	0.00000	0.00000	0.31812	33.0	1.96	0.05	0.02
a =5.102 Å	Li2	2b	0.00000	0.50000	0.66325				
b = 5.293 Å	Te1	2a	0.00000	0.00000	0.89667				
c = 8.988 Å	Te2	2b	0.00000	0.50000	0.08939				
	O1	4c	0.21217	0.65593	0.92734				
	O2	4c	0.21455	0.18574	0.06889				
	O3	4c	0.20975	0.68100	0.23479				

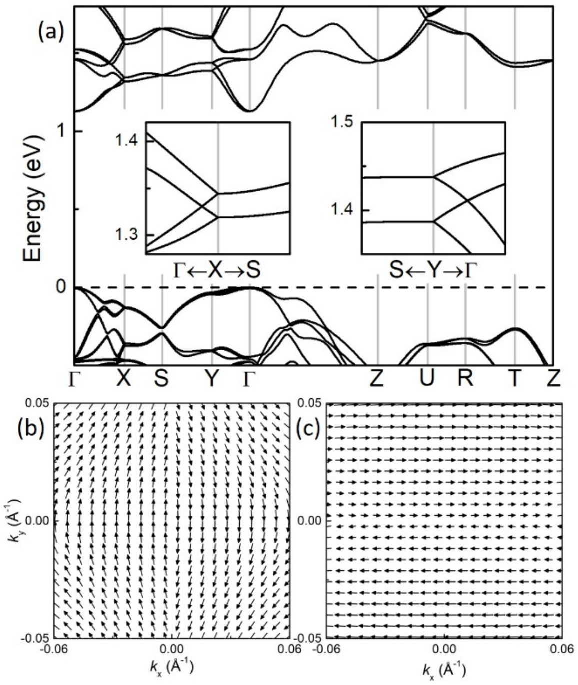

Supplementary Figure 5. Band structure and spin texture of $BiInS_3$. (a) Band structure of bulk $BiInS_3$. Inset: zoom-in band structure around the X and Y points. Spin textures in the k_z = 0 plane around the (b) X point and (c) Y point for the lowest conduction band. Note that the wave vector is measured from the X point for (b) and the Y point for (c).

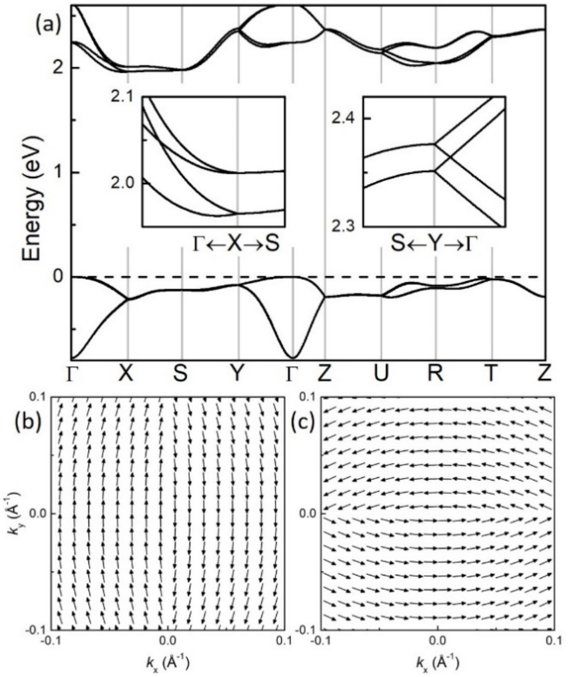

Supplementary Figure 6. **Band structure and spin texture of $LiTeO_3$.** (a) Band structure of bulk $LiTeO_3$. Inset: zoom-in band structure around the X and Y points. (b, c) Spin textures in the k_z = 0 plane around the X point (b) and Y point (c) for the lowest conduction band. Note that the wave vector k_x is measured from the X point for (b) and from the Y point for (c). CBM is about 0.01 Å^{-1} from the X point.

Supplementary Figs. 5 and 6 show the band structure and spin texture of $BiInS_3$ and $LiTeO_3$. We can see the similar spin textures around the X and Y points as compared with that for $BiInO_3$. For $BiInS_3$, the CBM is located at the Γ point. The CBM for $LiTeO_3$ is slightly shifted from the X point.

Supplementary Note 5.

Symmetry allowed terms up to cubic order in *k*

Supplementary Table 3. The symmetry allowed terms in the Hamiltonian within the $k_z = 0$ plane around the X point for crystals of space groups 28, 29, 31-34, 40, 41, 45, and 46 up to cubic order in *k*. The terms are classified according to their effect on PST in zero- and first-order perturbation for the wave function.

Order in wave vector k	Symmetry allowed terms (all preserve PST in zero order perturbation theory)	Terms which break PST in first order perturbation theory
**Linear**	$k_x\sigma_y, k_y\sigma_x, k_x\tau_y, k_x\tau_x\sigma_x, k_x\tau_z\sigma_z, k_y\tau_x\sigma_y$	$k_y\sigma_x, k_y\tau_x\sigma_y$
**Quadratic**	$k_x^2, k_y^2, k_xk_y\tau_x, k_x^2\tau_y\sigma_y, k_y^2\tau_y\sigma_y, k_xk_y\tau_y\sigma_x$	$k_xk_y\tau_x, k_xk_y\tau_y\sigma_x$
**Cubic**	$k_x^3\sigma_y, k_x^3\tau_y, k_x^3\tau_x\sigma_x, k_x^3\tau_z\sigma_z, k_y^3\sigma_x, k_y^3\tau_x\sigma_y, k_xk_y^2\sigma_y,$ $k_yk_x^2\sigma_x, k_xk_y^2\tau_y, k_xk_y^2\tau_x\sigma_x, k_xk_y^2\tau_z\sigma_z, k_yk_x^2\tau_x\sigma_y$	$k_y^3\sigma_x, k_y^3\tau_x\sigma_y, k_yk_x^2\sigma_x, k_yk_x^2\tau_x\sigma_y$

Supplementary Note 6.

Comparison between semiconductor quantum-well structures and non-symmorphic compounds

Supplementary Table 4. Comparison of PST and PSH properties in semiconductor quantum-well structures and non-symmorphic compounds.

	Semiconductor quantum well	Non-symmorphic compound
**Spin structure**	Persistent spin texture	Persistent spin texture
**Physical origin**	Balanced Rashba and linear Dresselhaus parameters	Enforced by non-symmorphic space group symmetry
**Deviation from PST**	Cubic in *k* Dresselhaus term	Linear in *k* perturbation term
**Precession frequency**	0.1–1 THz range	100 THz range
**PSH wavelength**	μm scale	nm scale

Supplementary references

1. Z. Wang, A. Alexandradinata, R. J. Cava, and B. A. Bernevig, Nature **532**, 189 (2016).
2. T. Bzdušek, Q. S. Wu, A. Rüegg, M. Sigrist, and A. A. Soluyanov, Nature **538**, 75 (2016).
3. R. Winkler, *Spin-Orbit Coupling Effects in Two-Dimensional Electron and Hole Systems*, Springer Tracts in Modern Physics (Springer, Berlin, 2003).
4. Q. Liu, X. Zhang, J. A. Waugh, D. S. Dessau, and A. Zunger, Phys. Rev. B **94**, 125207 (2016).
5. V. Kramer, Thermochim. Acta **86**, 291 (1985).
6. C. Lin, W. Cheng, Z. Luo, and G. Chai, J. Solid State Chem. **199**, 78 (2013).
7. A. Jain, S. P. Ong, G. Hautier, W. Chen, W. D. Richards, S. Dacek, S. Cholia, D. Gunter, D. Skinner, G. Ceder, and K. A. Persson. APL Mater. **1**, 011002 (2013). See also https://www.materialsproject.org/.
